# Employing Squeeze-and-Excitation Architecture in a Fine-Tuned Convolutional Neural Network for Magnetic Resonance Imaging Tumor Classification

**DOI:** 10.7759/cureus.80084

**Published:** 2025-03-05

**Authors:** Kian A Huang, Ahmad Alkadri, Neelesh Prakash

**Affiliations:** 1 Radiology, University of South Florida Health Morsani College of Medicine, Tampa, USA

**Keywords:** artificial intelligence in radiology, brain tumors, computer vision, deep learning, machine learning

## Abstract

In 2021, there were 182,520 cases of brain and central nervous system (CNS) cancers in the U.S. and 25,400 new cases of brain cancer in 2024. Early detection via magnetic resonance imaging (MRI) significantly improves patient outcomes. This study fine-tunes a residual neural network 50 version 2 (ResNet50V2), a convolutional neural network (CNN), with squeeze-and-excitation (SE) attention mechanisms to enhance MRI-based tumor classification compared to a base ResNet50V2 model. By incorporating SE blocks, the model improves feature prioritization, effectively distinguishing glioma (n = 901), meningioma (n = 913), pituitary tumor (n = 844), and no tumor (n = 438). Trained on a publicly available Kaggle dataset (N = 3,096), the proposed model achieved a 98.4% classification accuracy and an area under the receiver operating characteristic curve (AUC) of 0.999, outperforming the base model's 92.6% accuracy and 0.987 AUC. Statistically significant improvements were observed in meningioma (p = 0.013) and pituitary tumor (p = 0.015) classification accuracy, highlighting the SE model's superior ability to differentiate tumor types. SE attention mechanisms enhance diagnostic precision by addressing feature extraction limitations and long-range dependencies in medical imaging. However, challenges such as dataset size constraints, overfitting risks, and potential representation bias remain. Future research will focus on expanding dataset diversity, exploring vision transformers (ViTs) for improved feature extraction, and employing generative adversarial networks (GANs) for data augmentation.

## Introduction

Brain and central nervous system (CNS) tumors, such as gliomas, meningiomas, and pituitary adenomas, can have drastic physical and psychological consequences for patients, often creating life-altering challenges, especially when diagnosed at an advanced stage; in 2021, there were 182,520 cases of brain and CNS cancers in the United States with approximately 25,400 new cases of brain and CNS cancer in 2024, as well as 18,760 brain and CNS cancer-related deaths, accounting for 3.1% of all cancer deaths [1-3]. Early detection of brain tumors, especially on magnetic resonance imaging (MRI), has been shown to improve patient prognosis, increasing the likelihood of survival, but are difficult to detect in earlier stages due to subtle findings on imaging [3,4]. Given the heterogeneous nature of brain tumor types and their diverse presentations, accurate and timely detection is crucial [2,5]. The application of artificial intelligence (AI), namely, deep learning through convolutional neural networks (CNNs), for MRI tumor diagnosis has been promising, often showing a reduction in error in early diagnosis [5]. CNNs are deep learning models that analyze images by detecting patterns and features, such as edges, textures, and shapes. They process images in layers, first identifying basic structures and then combining them to recognize complex patterns, like tumors or abnormalities. Thus, there is great potential for CNNs to utilize MRIs to provide more accurate and timely brain tumor diagnoses.

Image recognition on MRI has been demonstrated before on various CNN architectures, such as Residual Network 50 Version 2 (ResNet50V2), Visual Geometry Group 16 (VGG16), and Inception [6]. ResNet50V2, with its innovative use of residual connections, addresses vanishing gradient issues and is a reliable backbone for transfer learning in medical imaging [7]. Compared to other models, such as VGG16, ResNet50V2 offers deeper feature extraction while maintaining computational efficiency [6, 7]. While transformer-based models have shown promise in medical imaging, ResNet50V2 was chosen for its balance between performance and efficiency, making it well-suited for MRI tumor classification without the extensive data requirements of transformers [6,7]. Multi-classification brain tumor models on similar datasets have been created using ResNet50V2 but seem to plateau around a 95% testing accuracy [8,9]. Current CNN architectures may be limited in capturing long-range dependencies and struggle to focus on relevant features in the data, potentially undermining their accuracy and representational power [10]. To overcome this challenge, we propose an innovative approach, using a fine-tuned ResNet50V2 model with a squeeze-and-excitation (SE) attention mechanism to improve feature prioritization on brain tumor imaging classification [10]. SE blocks enhance feature maps by adaptively recalibrating channel-wise feature responses, enabling models to concentrate on the most informative regions of an image [10]. This report validates the use of a fine-tuned ResNet50V2 model with an SE framework to enhance classification accuracy. It also serves as a proof-of-concept of the technical deployment of a ResNet50V2-SE model with exceptional performance in classifying brain tumor imaging composed of meningiomas, gliomas, pituitary tumors, and tumor-free MRIs and its comparison to a baseline ResNet50V2 model without attention modulation.

## Technical report

Data collection and preprocessing

This study utilized a publicly available dataset from Kaggle, comprising 3,096 256 x 256-sized T1 and T2 MRI brain tumor images. The images were categorized into four groups: meningioma tumor, pituitary tumor, glioma tumor, and no tumor. To ensure diverse representation, the images included coronal, transverse, and sagittal views. To prepare the dataset for analysis, preprocessing was performed to standardize the dimensions and quality of the images. The class distribution of the dataset was as follows: glioma (n = 901), meningioma (n = 913), no tumor (n = 438), and pituitary tumor (n = 844). To maintain consistency and compatibility with ResNet50V2 initial layer input, all images were resized to 224 × 224 pixels while preserving their original aspect ratios. The dataset was randomly shuffled and divided into three subsets: 80% for training, 10% for validation, and 10% for testing. To make the model more robust and reduce overfitting, data augmentation was applied to the training set via horizontal flipping of images, which effectively increased the dataset size and introduced variability. 

Model development and architecture

The model was built upon the ResNet50V2 architecture, a widely used convolutional neural network trained on the ImageNet dataset [6]. To adapt this pre-trained model for tumor classification, transfer learning was employed. The initial layers of ResNet50V2, which extract general features, were frozen to retain their pre-trained weights. The deeper layers, starting from the “conv5_block1_preact_bn” layer, were unfrozen to allow the model to learn specific features relevant to the MRI tumor dataset. We allowed the deeper, more specialized layers of the model to be updated during training as these layers are closer to the final output and are responsible for recognizing highly detailed and task-specific features, such as subtle patterns in MRI images, without losing the general knowledge it gained from being pre-trained on a broader set of images

To enhance the model’s ability to focus on critical regions within the images, attention mechanisms were incorporated. An SE block was added to refine the importance of features at the channel level, enabling the model to prioritize the most diagnostically relevant information. After the ResNet50V2 base, the model architecture included three convolutional layers with 32, 64, and 128 filters, respectively. Each of these layers was followed by batch normalization, which stabilized training by normalizing activations within each mini-batch. This reduced internal covariate shift, accelerated convergence, and provided regularization to help prevent overfitting. Max pooling was applied after each convolutional layer to reduce the spatial dimensions, which preserved computational efficiency while retaining essential features.

The fully connected layers included a dense layer with 128 neurons and ReLU (rectified linear unit) activations, which introduced non-linearity to the model and prevented the vanishing gradient problem. ReLU’s ability to set negative values to zero allowed the model to focus on learning meaningful feature patterns, which is particularly valuable for detecting subtle distinctions in medical images. To address overfitting, dropout layers were added, randomly deactivating neurons during training to promote diverse feature learning and improve generalization. The final layer used a softmax activation function to classify the images into one of the four tumor categories. The model was compiled using the Adam optimizer with an initial learning rate of 0.0001, while categorical cross-entropy was selected as the loss function to address the multi-class classification task. During training, early stopping halted the process when no further improvement was observed (epochs = 100, batch size = 16), ensuring efficient resource use. In addition, the learning rate was reduced when validation loss plateaued, allowing for steady progress during optimization.

Evaluation metrics

The performance of the model was evaluated using accuracy, precision, recall, F1 score, and area under the receiver operating characteristic curve (AUC). Accuracy quantified the overall proportion of correctly classified images across all categories. Precision measured the percentage of true positive predictions among all positive predictions made by the model (true and false positives). Recall, also referred to as sensitivity, quantified the model's ability to correctly identify actual positive instances among all instances (true positives and false negatives). The F1 score, a harmonic mean of precision and recall, provided a single, balanced metric to assess the model’s performance, which is particularly valuable for scenarios involving imbalanced datasets. AUC was calculated for each tumor class and the model as a whole. The AUC score measures the model's ability to distinguish between classes across different classification thresholds, with a value of 1.0 indicating perfect classification.

A confusion matrix was generated to further analyze the model’s classification performance. This matrix provided a detailed breakdown of true positives, true negatives, false positives, and false negatives for each class, offering insights into areas where the model performed well and where it struggled. The confusion matrix is particularly useful for understanding the specific types of errors the model made, such as misclassifications between similar tumor types, and serves as a valuable tool for clinicians to interpret the model's behavior in practical applications.

Model performance 

**Figure 1:**
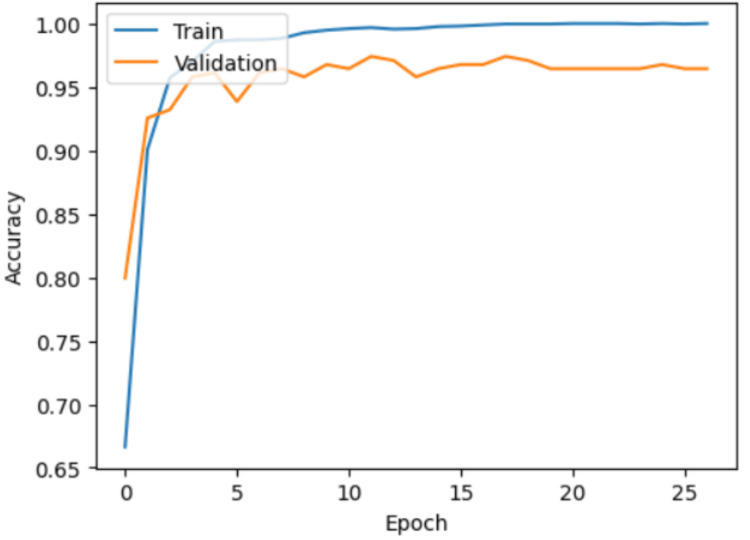
Model training and validation accuracy over time while learning the MRI tumor dataset.

**Figure 2:**
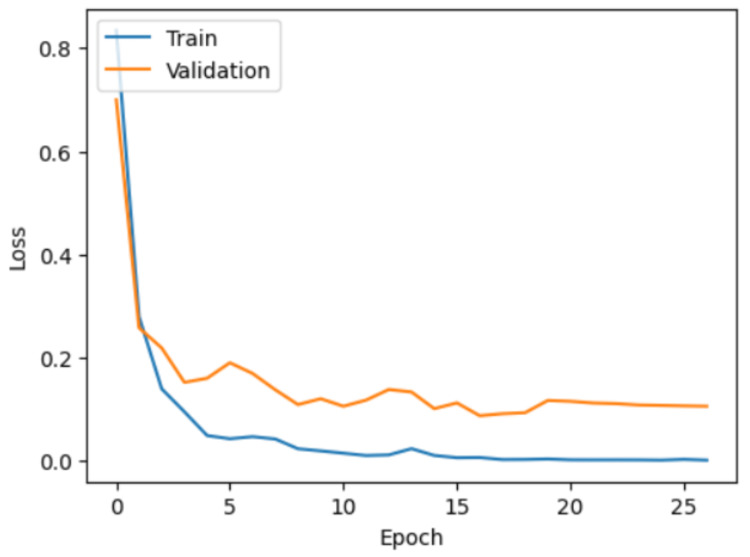
Model training and validation loss over time while learning the MRI tumor dataset.

The performance of the model was evaluated using the training, validation, and test datasets. The model used the training data for learning, with validation testing occurring immediately after each learning epoch. The test set was introduced only after training was complete and consisted of images that the model had never seen before. The training process achieved convergence, as illustrated in the accuracy and loss graphs in Figures 1-2. Throughout 27 epochs, the model's training accuracy steadily improved, surpassing 95% by epoch 5 and reaching nearly 100% by the final epoch. Validation accuracy followed a similar trajectory, stabilizing above 95% (Figure 1). Concurrently, both training and validation losses demonstrated a consistent downward trend, reflecting effective model optimization and a reduced likelihood of overfitting (Figure 2).

The accuracy graph (Figure 1) highlights the model's ability to correctly classify images over time, with training and validation accuracy trends providing insight into learning stability. Meanwhile, the loss graph (Figure 2) indicates how well the model minimizes errors, with decreasing loss values signaling improved performance and convergence toward an optimal solution.

**Figure 3:**
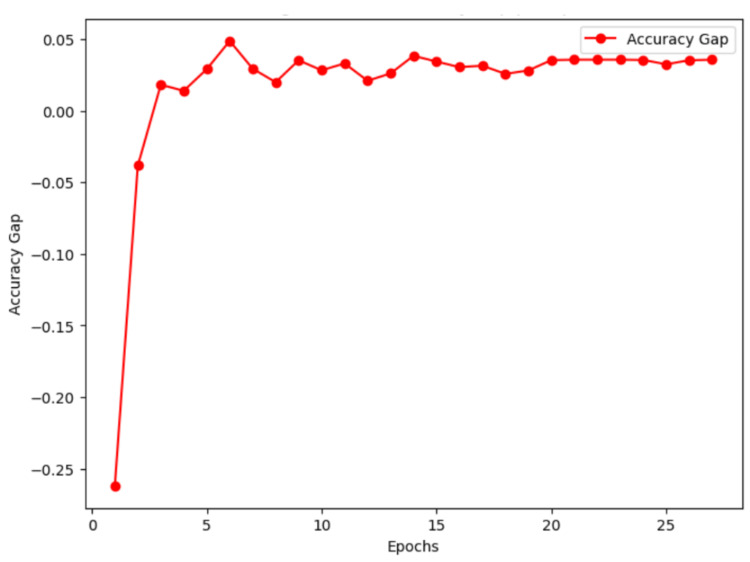
Training vs. validation accuracy gap across epochs Accuracy gap between training and validation over 27 epochs. The gap stabilizes at 0.036 in the final epoch, indicating minimal overfitting and strong generalization performance.

To further evaluate overfitting, we analyzed the gap between training and validation accuracy across epochs, as shown in Figure 3. While an initial discrepancy was observed in the early stages of training, the gap quickly stabilized, remaining around 0.036 in the final epochs. This minimal gap suggests that the model effectively generalized without excessive overfitting.

Table 1 summarizes the SE model's classification performance on the holdout test for each tumor category using precision, recall, F1 score, and AUC scores. The overall model accuracy and AUC were also calculated, demonstrating an accuracy of 98.4%, and an AUC of 0.999, demonstrating excellent classification performance and discrimination. The SE model outperformed the base ResNet50V2 model in the vast majority of performance categories. Table 2 demonstrates the base model performance without attention modulation with an accuracy of 92.6% and an AUC of 0.987. A two-proportion Z-test was conducted to compare the test overall and subclassification accuracies between each model. No statistically significant differences were found between overall, glioma, and normal classification accuracies. However, the SE model had higher testing accuracies in meningiomas and pituitary tumors compared to the base model (p = 0.013 and p = 0.015, respectively).

**Table 1:** Squeeze-and-excitation model performance by tumor classification Overall and individual classification results from model performance during testing. AUC = area under the receiver operating characteristic curve

Tumor Category	Precision	Recall	F1 score	AUC	Accuracy
Glioma tumor	0.99	0.97	0.98	0.998	0.98
Meningioma tumor	0.97	0.99	0.98	0.997	0.98
Normal (no tumor)	0.97	0.97	0.97	0.999	0.97
Pituitary tumor	1	1	1	1	1
Overall	N/A	N/A	N/A	0.999	0.98

**Table 2:** Base ResNet50V2 model performance by tumor classification Overall and individual classification results from model performance during testing. AUC = area under the receiver operating characteristic curve.

Tumor category	Precision	Recall	F1 score	AUC	Accuracy
Glioma tumor	0.97	0.87	0.92	0.986	0.92
Meningioma tumor	0.87	0.91	0.89	0.970	0.89
Normal (no tumor)	1	0.98	0.99	1	0.99
Pituitary tumor	0.89	0.96	0.93	0.992	0.93
Overall	N/A	N/A	N/A	0.987	0.93

**Figure 4:**
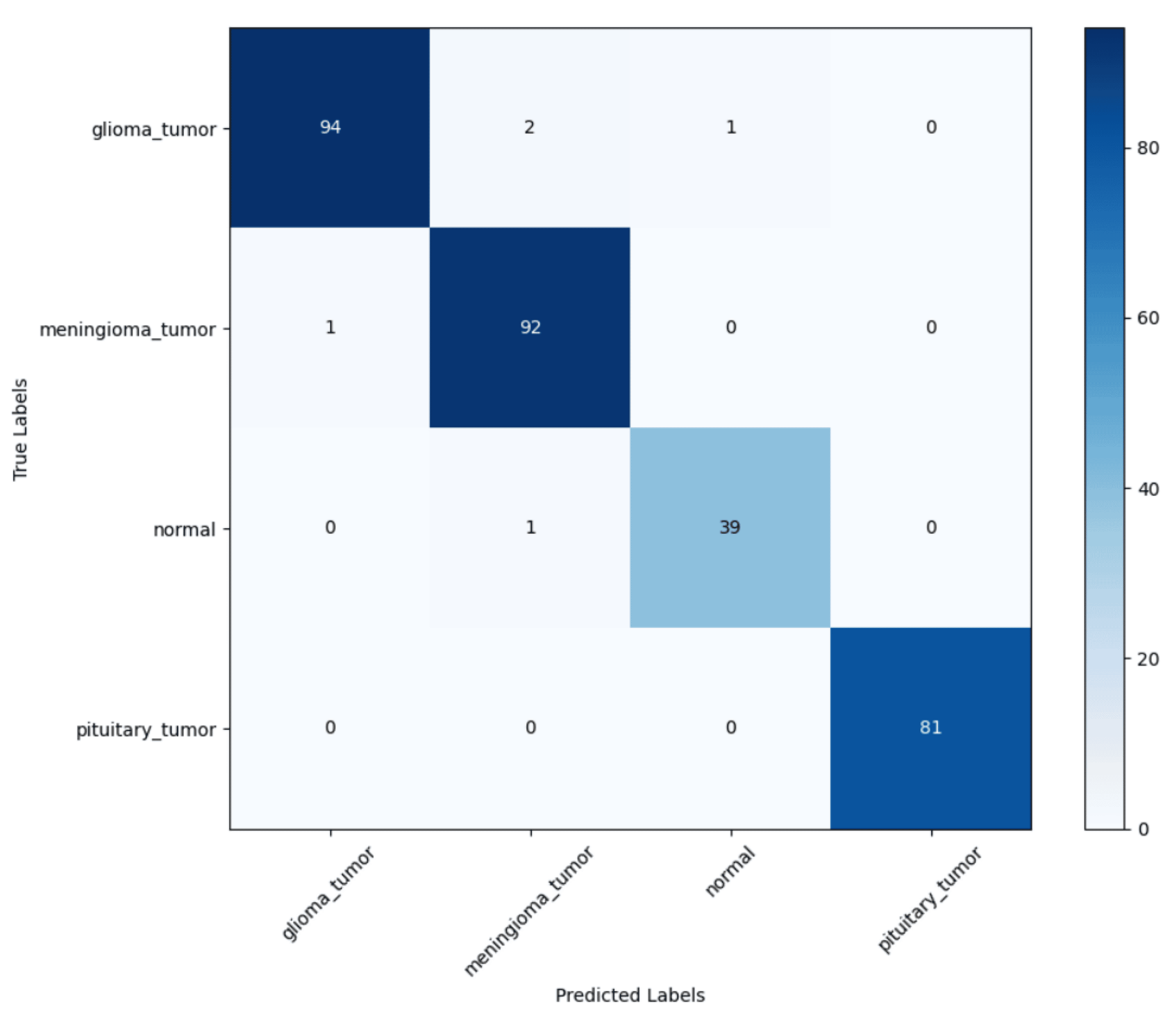
Confusion matrix of model testing performance Matrix demonstrating which errors the model made during testing by classification.

The confusion matrix, depicted in Figure 4, provides additional insight into the model's classification accuracy. It visually represents the number of correct and incorrect predictions for each class. The model demonstrated exceptional performance in classifying pituitary tumors, achieving perfect precision, recall, and F1 scores with no misclassifications. Minor misclassifications were observed in other classes, such as glioma and meningioma tumors, where a small number of cases were incorrectly predicted as belonging to adjacent categories. 

**Figure 5:**
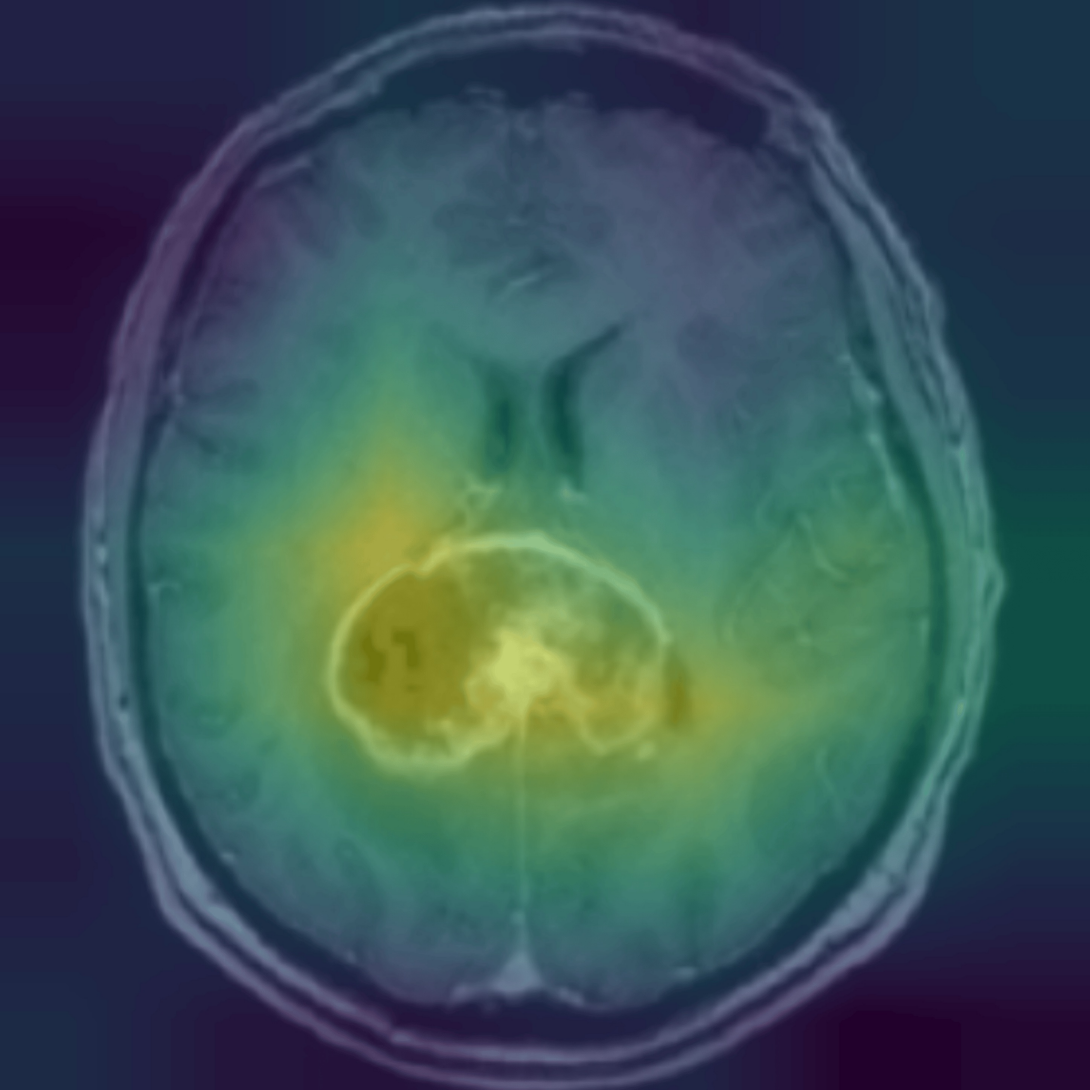
Grad-CAM visualization of a glioma MRI scan Grad-CAM heatmap highlighting the regions of interest in a sample glioma MRI scan. The model primarily focuses on the tumor-affected area, demonstrating its ability to identify key pathological features for classification.

To further interpret the model’s decision-making process, we utilized gradient-weighted class activation mapping (Grad-CAM), as shown in Figure 5, demonstrating the model's identification approach in a sample glioma MRI. Grad-CAM highlights the regions in the MRI scan that contributed most to the model’s classification decisions. The visualization demonstrates that the model primarily focuses on tumor-affected regions, reinforcing its ability to differentiate pathological features from normal brain structures. This interpretability is crucial in medical imaging applications as it enhances trust in AI-driven diagnostics and provides insights into potential misclassifications.

## Discussion

This study introduces an enhanced model that fine-tunes ResNet50V2 with SE attention mechanisms, which dynamically recalibrates channel-wise feature responses to improve tumor identification. The fine-tuned ResNet50V2-SE model achieved exceptional classification performance, with an overall accuracy of 98.4% and an AUC of 0.999, demonstrating a pronounced ability to separate and distinguish between glioma, meningioma, pituitary tumors, and non-tumor cases. As seen in Table 1, the model’s perfect precision, recall, and F1-score for pituitary tumor classification are likely due to the distinct presentation of pituitary tumors on MRI. The ResNet50V2-SE model also performed well on an imbalanced dataset, demonstrating high precision and recall across all tumor classes. Minor misclassifications observed in the confusion matrix suggest that glioma and meningioma tumors share overlapping imaging characteristics, but the overall error rate remained minimal. To further analyze model interpretability, heatmaps were generated using Grad-CAM to visualize the regions within MRI scans that contributed most significantly to classification decisions. These heatmaps demonstrated that the SE model effectively concentrated on tumor-specific areas while minimizing background noise. The strong localization of tumor features in heatmaps further supports model transparency and model validity of the SE-enhanced model by providing visual confirmation of its decision-making process.

A key enhancement introduced in this study was the integration of SE attention mechanisms, which significantly improved classification performance over the base ResNet50V2 model. Previous studies utilizing ResNet50V2 and other CNN architectures for MRI tumor classification have encountered challenges such as plateauing accuracy and difficulty in capturing long-range dependencies [8,9]. By incorporating SE blocks, this study addressed these limitations, allowing the model to enhance feature prioritization and improve overall classification accuracy. Comparative analysis revealed that the SE model outperformed the base ResNet50V2 model, which achieved an accuracy of 92.6% and an AUC of 0.987. Notably, the SE model demonstrated statistically significant improvements in meningioma (p = 0.013) and pituitary tumor (p = 0.015) classification accuracy, underscoring its ability to extract diagnostically relevant features more effectively.

To evaluate potential overfitting, the gap between training and validation accuracy was analyzed throughout training. While an initial discrepancy was observed, the gap quickly stabilized at approximately 0.036 in the final epochs, suggesting effective generalization without excessive overfitting. In addition, training and validation losses demonstrated a consistent downward trend, reinforcing the model’s stability.

Despite its strong performance, a potential limitation of this model is its reliance on a relatively small and specific dataset without external validation, which may not fully capture the variability present in clinical scenarios. This constraint can lead to overfitting, where the model performs well on training data but struggles to generalize to new, unseen data [11]. Overfitting remains a common challenge in deep learning, as models may unintentionally focus on noise within the dataset or memorize unique features rather than learn generalizable patterns [11]. In addition, like all CNNs, this model may be susceptible to representation bias and domain shift when tested on external datasets [12]. Representation bias arises when the training data does not adequately reflect the diversity of the target population, leading to potential misclassifications in underrepresented subgroups [12]. Domain shift refers to differences between the training data distribution and real-world data distribution, which can impact clinical applicability [12].

Future efforts will focus on expanding the dataset by incorporating images from multiple sources, such as various hospitals and clinics. This approach aims to capture a broader spectrum of variability, thereby enhancing the model's generalizability and robustness. In addition to dataset expansion, exploring alternative deep learning architectures beyond traditional CNNs may offer performance improvements. Vision transformers (ViTs) have shown promise in medical image analysis, demonstrating a lower tendency for hidden stratification compared to CNNs [13]. Similarly, generative adversarial networks (GANs) have been utilized to augment medical imaging data, potentially enhancing model training by providing more diverse examples within the dataset without the burdens of traditional data collection [14]. Future research may also explore hybrid architectures that combine the strengths of CNNs and ViTs, optimizing both local and global feature extraction in MRI-based tumor classification. By refining attention-based deep learning approaches and expanding validation efforts, this study paves the way for more accurate, scalable, and clinically applicable AI-driven tumor detection models.

## Conclusions

This study demonstrates the effectiveness of integrating SE attention mechanisms with transfer learning on a pretrained ResNet50V2 model for MRI-based brain tumor classification. The SE-enhanced model achieved 98.4% accuracy and an AUC of 0.999, outperforming the baseline ResNet50V2 model (92.6% accuracy, AUC 0.987) with statistically significant improvements in meningioma and pituitary tumor classification (p = 0.013 and p = 0.015, respectively). Heatmap analysis confirmed the model’s ability to focus on diagnostically relevant tumor regions, reinforcing its clinical and research potential.

Despite strong performance, this study relied solely on a holdout test set without external dataset validation, which may limit generalizability. Real-world clinical applications require models that perform robustly across diverse populations, and a lack of external validation poses risks of representation bias and domain shift. Future research must incorporate multi-institutional data collection to create diverse external datasets, ensuring broader applicability and improved performance across different imaging protocols, scanner types, and patient demographics. In addition, exploring architectures like ViTs and GANs may further enhance generalizability and mitigate dataset limitations. Validating this model on independent datasets will be a critical next step in translating AI-driven tumor classification into clinical practice.
